# Bioluminescence Imaging to Track *Bacteroides fragilis* Inhibition of *Vibrio parahaemolyticus* Infection in Mice

**DOI:** 10.3389/fcimb.2017.00170

**Published:** 2017-05-11

**Authors:** Zhengchao Li, Huimin Deng, Yazhou Zhou, Yafang Tan, Xiaoyi Wang, Yanping Han, Yangyang Liu, Ye Wang, Ruifu Yang, Yujing Bi, Fachao Zhi

**Affiliations:** ^1^Guangdong Provincial Key Laboratory of Gastroenterology, Department of Gastroenterology, Institute of Gastroenterology of Guangdong Province, Nanfang Hospital, Southern Medical UniversityGuangzhou, China; ^2^State Key Laboratory of Pathogen and Biosecurity, Beijing Institute of Microbiology and EpidemiologyBeijing, China; ^3^Guangzhou ZhiYi Biotechnology Co. Ltd.Guangzhou, China

**Keywords:** *Bacteroides fragilis*, *Vibrio parahaemolyticus*, real-time cell analysis, bioluminescence, *in vivo* imaging

## Abstract

*Bacteroides fragilis* is an anaerobic, Gram-negative, commensal bacterium of the human gut. It plays an important role in promoting the maturation of the immune system, as well as suppressing abnormal inflammation. Many recent studies have focused on the relationship between *B. fragilis* and human immunity, and indicate that *B. fragilis* has many useful probiotic effects. As inhibition of intestinal pathogens is an important characteristic of probiotic strains, this study examined whether *B. fragilis* could inhibit pathogenic bacteria. Results showed that *Vibrio parahaemolyticus* was inhibited by *B. fragilis in vitro*, and that *B. fragilis* could protect both RAW 264.7 and LoVo cells from damage caused by *V. parahaemolyticus*. Using *in vivo* imaging, we constructed a light-emitting *V. parahaemolyticus* strain and showed that *B. fragilis* might shorten the colonization time and reduce the number of *lux*-expressing bacteria in a mouse model. These results provide useful information for developing *B. fragilis* into a probiotic product, and also indicate that this commensal bacterium might aid in the clinical treatment of gastroenteritis caused by *V. parahaemolyticus*.

## Introduction

*Bacteroides fragilis* is a commensal bacterium of the human gut (Mazmanian and Kasper, 2006). Recently, many studies have found that *B. fragilis* plays an important role in promoting the maturation of the immune system, suppressing abnormal inflammation, and improving the structure of intestinal microflora (Mazmanian et al., 2008; Round et al., 2011). Further, some studies have shown that *B. fragilis* can reduce the symptoms of autism in mice, even without the help of polysaccharide A (Hsiao et al., 2013). Therefore, *B. fragilis* is an extremely promising candidate probiotic species. Most currently-available probiotics, such as those based on *Lactobacillus* and *Bifidobacterium* species, play a role in promoting human health. Beneficial effects of these products include relieving constipation, helping digestion, enhancing immunity, relieving lactose intolerance, reducing dysbacteriosis resulting from antibiotic overuse, and treating diarrhea caused by pathogenic intestinal bacteria (Vanderhoof, 2001; Mazlyn et al., 2013; Ashraf and Shah, 2014; Pandey et al., 2015). Therefore, in this study we aimed to examine the probiotic efficacy of *B. fragilis* and determine its ability to inhibit pathogenic bacteria.

*Vibrio parahaemolyticus* is the leading cause of bacterial seafood-borne gastroenteritis worldwide. Symptoms of *V. parahaemolyticus* infection range from mild self-limiting diarrhea to severe cholera-like abdominal pain, diarrhea, abdominal cramps, nausea, vomiting, and fever. Feces is watery in most patients, but may contain blood in severe cases. In addition, the immunity of patients following infection is usually so weakened that repeat infection is common (Wang et al., 2015; Odeyemi, 2016). Multiple virulence factors have been described for *V. parahaemolyticus*, including a heat-resistant hemolysin, cellular adhesion, urease production, lipopolysaccharide, a type III secretion system, and mucinase production (Broberg et al., 2011; Ham and Orth, 2012); however, the exact mechanism of pathogenesis has not been elucidated.

The aim of this study was to evaluate the probiotic effects of *B. fragilis* against *V. parahaemolyticus* in intestinal epithelial cells and in an *in vivo* mouse model. Real-time cell analysis (RTCA) technology was used for *in vitro* tissue culture experiments, while bioluminescence imaging was used to track bacterial cells in the mouse assays. In the RTCA system, microelectrodes are present in the bottom of the cell culture plates. Cell growth, including changes in cell number, morphology, and degree of attachment, in the plates changes the resistance of the microelectrodes. This allows growth status and the integrated state of all the cells to be transferred into electrical signals, which are recorded and presented by computers. This technology allows continuous monitoring of an experiment without the addition of any detection reagent or destructive sampling (Roshan Moniri et al., 2015; Valdes et al., 2015).

Although, mice are not natural hosts of *V. parahaemolyticus*, mouse models are still common used for examining the pathogenesis of *V. parahaemolyticus in vivo* (Hiyoshi et al., 2010; Yang et al., 2013; Whitaker et al., 2014). In addition to the mouse model, it has been reported that infant rabbits infected with *V. parahaemolyticus* develop severe diarrhea and enteritis that mimic the main clinical and pathologic manifestations of the disease in humans, so the infant rabbit model is also a good choice for the research of *V. parahaemolyticus* (Ritchie et al., 2012; Zhou et al., 2013, 2014). However, we found that infant rabbits could not tolerate frequent and deep anesthesia, so this model will be subject to our experiment. In traditional gavage infection assays of *V. parahaemolyticus* in animals, infection is confirmed and monitored by fecal pathogen count, observation of symptoms in experimental animals, and pathological examination of intestinal injury. While previous studies have found that *Lactobacillus* can prevent *V. parahaemolyticus* infection in mice (Yang et al., 2013), technical limitations of traditional methods mean that experiments cannot truly determine the number of bacteria in the intestine or identify the site of colonization. However, bioluminescence imaging allows noninvasive monitoring of bacteria in animals. In 1995, Contag et al. introduced plasmids containing the *lux* system into *Salmonella* for evaluation of virulence in mice, thereby pioneering the use of bioluminescent systems to tag bacteria for *in vivo* imaging experiments in small animals (Contag et al., 1995). Experimental animals were then infected with the *lux*-expressing strain. Animals were then anesthetized, and *in vivo* imaging was performed using a living body imaging system (Brock et al., 2008). In the current study, the *lux* luminescence system was introduced into *V. parahaemolyticus*, resulting in a self-luminous *lux* strain. In this way, we investigated the proliferation, translocation, and colonization of *lux* strains *in vivo* by real-time kinetic tracing.

## Materials and methods

### Bacterial strains and culture conditions

*B. fragilis* strain ZY-312 was provided from Zhiyi Biological Technology Co., Ltd. (Guangzhou, Guangdong province, China; Deng et al., 2016; Wang et al., 2017). The strain was cultured in trypticase soya broth (TSB; Oxoid, Basingstoke, UK) supplemented with 5% fetal bovine serum (MP Biomedicals, USA) at 37°C for ~16 h in an anaerobic incubator (Bugbox, Ruskinn). Ten-fold serial dilutions of the overnight cultures were plated on trypticase soya agar (TSA; Oxoid, Basingstoke, UK) containing 5% fresh sheep blood to determine the initial bacterial concentrations [presented as colony forming units (cfu/ml)]. Wild-type *V. parahaemolyticus* strain rimd2210633 (Makino et al., 2003), an ampicillin-resistant clinical isolate originally isolated from a patient with diarrhea, was obtained from the Academy of Military Medical Science, Beijing, China. It was cultured in 3.5% NaCl Luria-Bertani broth (LB; Oxoid, Basingstoke, UK) at 37°C in a shaking incubator for ~8 h.

### The oxford cup assay

Bacterial cells were harvested in log phase (*B. fragilis* at 16 h, *V. parahaemolyticus* at 8 h) by centrifugation (4,000 rpm, 10 min), washed twice with sterile phosphate-buffered saline (PBS), and then diluted in PBS to OD_600_ = 1.0 (corresponding to ~10^9^ cfu/ml). The bacterial cell density was adjusted to the target concentration with PBS where necessary. *B. fragilis* culture supernatant was filtered using a 0.22-μm filter membrane (Millex) to ensure all bacterial cells had been removed. A 1-ml aliquot of *B. fragilis* at a concentration of 10^9^ cfu/ml was lysed by sonication and then filtered through a 0.22-μm filter membrane. Aliquots (0.2 ml) of *V. parahaemolyticus* at a concentration of 10^5^ cfu/ml were then spread evenly on the surfaces of LB plates, and 3–4 Oxford cups were placed onto each plate. Next, 250-μl volumes of PBS, sterile TSB, filter-sterilized *B. fragilis* supernatant, *B. fragilis* lysate, and resuspended *B. fragilis* at a concentration of 10^9^ cfu/ml were individually added to the Oxford cups. The plates were then incubated at 4°C for 12 h and then 36°C for 12 h prior to observation.

### RTCA pathogen inhibition assay

Tumor-derived human colonic epithelial LoVo cells and tumor-derived mouse macrophage RAW 264.7 cells were obtained from the Academy of Military Medical Science. LoVo cells were cultured in Dulbecco's Modified Eagle Medium/nutrient mixture F12 (DMEM-H/F12) (1:1) (Gibco, USA) supplemented with 10% fetal bovine serum (MP Biomedicals, USA). RAW 264.7 cells were cultured in DMEM medium (Gibco) supplemented with 10% fetal bovine serum (MP Biomedicals). Both cell lines were cultured under a moist atmosphere in a 5% CO_2_ incubator (MCO-18AIC SANYO, Japan) at 37°C. Cells were diluted to a density of 2 × 10^5^ cells/ml in their respective culture media. For the RTCA assays (RTCA iCELLigence, ACEA Biosciences, USA), 150 μl of culture media were added to each well of the RTCA plates, which were then inserted into the station to obtain baseline measurements. A 300-μl volume of cell suspension (2 × 10^5^ cells/ml) was added to each well, and plates were incubated in a 5% CO_2_ incubator at 37°C for 12–14 h. Following incubation, the culture medium was aspirated from all wells. A 300-μl volume of fresh culture medium was added to all control and *V. parahaemolyticus*-only treatment wells, while 300 μl of culture medium containing *B. fragilis* [10^8^ cfu/ml; multiplicity of infection (MOI) = 500] were added to *B. fragilis*-only and *B. fragilis* + *V. parahaemolyticus* treatment wells. Plates were incubated for a further 3 h before 30 μl of culture medium containing *V. parahaemolyticus* (10^8^ cfu/ml; MOI = 50) were added to *V. parahaemolyticus* and *B. fragilis* + *V. parahaemolyticus* treatment wells. The bacteria and cells were then co-incubated for another 3 h. Cells were monitored throughout the experiment using the xCELLigence system according to the manufacturer's instructions. At the end of the experimental period, cell morphologies were observed and photographed using a light microscope. The experiments were repeated three times.

### Construction of a bioluminescent *V. parahaemolyticus* strain

Plasmid pXEN-*lux*CDABE, was obtained from the Academy of Military Medical Science, Beijing, China. The plasmid encodes luciferase and substrate (Fatty Aldehydes). The luciferase and substrate, with the participation of oxygen and ATP, produce long chain fatty acids and release photons (450~490 nm) that can be detected. The plasmid also confers resistance to kanamycin. Wild-type *V. parahaemolyticus* was transformed with pXEN-*lux*CDABE by electroporation. 10^9^ cfu *V. parahaemolyticus* and 50 ng plasmid were added into 0.1 ml electroporation buffer (14% sucrose, 1 mM EDTA, 1 mM Hepes, pH 7.0) and placed in an ice-water mixture for 10 min before electroporation (25 μF, 200 Ω, 2.0 Kv). The resulting transformants were cultured on thiosulfate-citrate-bile salts-sucrose (TCBS) agar plates containing kanamycin (50 μg/ml), and then *lux*-expressing clones were selected using a NightOWL II LB983 imaging system (Berthold Technologies, Bad Wildbad, Germany). A *lux*-expressing clone was transferred to a fresh TCBS-kanamycin (50 μg/ml) agar plate (TCBS agar, LandBridge Beijing, China) for enrichment culture.

### Growth analysis of the wild-type and bioluminescent *V. parahaemolyticus* strains

The wild-type and *lux*-expressing *V. parahaemolyticus* strains were cultured in 5 ml of LB broth and LB broth supplemented with kanamycin (50 μg/ml), respectively, at 37°C in a shaking incubator for ~8 h. Optical density at 600 nm was measured every 2 h, and the resulting growth curves were plotted.

### Lactate dehydrogenase (LDH) cytotoxicity assay

LoVo cells were harvested and seeded at a density of 2 × 10^4^ cells/well into 96-well plates and then incubated overnight (12–14 h) at 37°C in a CO_2_ incubator. Cells were then infected with either the *lux*-expressing or wild-type *V. parahaemolyticus* strain at a MOI of 50, and the plates were incubated for 4 h at 37°C in a cell incubator. An LDH Cytotoxicity Detection Kit (Roche, USA) was used to measure the LDH released from damaged cells according to the manufacturer's recommendations. The released LDH was detected using a spectrophotometer (Promega, Madison, WI, USA) at OD_600_. Eight replicates were performed for each treatment, and the experiment was repeated three times.

### RTCA analysis of wild-type and bioluminescent *V. parahaemolyticus* strains

LoVo cells were harvested and diluted to a density of 2 × 10^5^ cells/ml in fresh culture medium. A total of 150 μl of culture medium were added to each well of RTCA plates, which were then inserted into the station to determine baseline measurements. A 300-μl volume of LoVo cell suspension was added to each well, and plates were incubated in a moist atmosphere in a 5% CO_2_ incubator at 37°C for 12–14 h. The culture medium was then aspirated from all wells, and a 300-μl volume of culture medium containing wild-type *V. parahaemolyticus* (1 × 10^7^ cfu/ml) was added to each of the *V. parahaemolyticus* treatment wells (MOI = 50). A 300-μl volume of culture medium containing *lux*-expressing *V. parahaemolyticus* (1 × 10^7^cfu/ml) was added to each of the *lux*-*V. parahaemolyticus* treatment wells (MOI = 50), and the plates were incubated for a further 12 h. Cells were monitored throughout the experimental period using the xCELLigence system.

### Bioluminescence imaging of infected animals

All animal experiments were conducted using 6-week-old female BALB/c mice raised under specific pathogen-free conditions, provided by the Small Animal Breeding Center, Academy of Military Medical Sciences. All animal experiments were conducted in accordance with the Guidelines for the Welfare and Ethics of Laboratory Animals of China, and were approved by the Committee of the Welfare and Ethics of Laboratory Animals, the Academy of Military Medical Science, Beijing, China (NO. SCXK-2015-01070021). Mice were fasted for 4 h prior to gavage. Twenty-four hours prior to each experiment, mice were randomly divided into two groups (control and treatment groups), marked with a unique number by ear-tag, and then gavaged with 0.2 ml of streptomycin (100 mg/ml).

The *lux*-expressing *V. parahaemolyticus* strain was cultured in LB medium supplemented with kanamycin (50 μg/ml) for 8 h at 37°C with shaking. The cell concentration was adjusted to 3 × 10^10^ cfu/ml with PBS, and all mice were gavaged with 0.3 ml of bacterial suspension. At 3 h post-gavage, mice were anesthetized with 3% isoflurane for 10 min, and *in vivo* imaging was carried out using the NightOWL II LB983 imaging system. *B. fragilis* was cultured in TSB supplemented with 5% fetal bovine serum at 37°C for ~16 h in an anaerobic incubator. Once revived, mice in the treatment group were gavaged with 0.3 ml of *B. fragilis* cell suspension (3 × 10^10^ cfu/ml), while those in the control group were gavaged with 0.3 ml of PBS. At 10 h post-inoculation, *in vivo* imaging was performed for the two groups, and the gavage step was repeated. *In vivo* imaging was then performed every 4 h until the bioluminescence was completely undetectable in one group. Results were analyzed using IndiGO software (Berthold Technologies, Germany). Mice were imaged in the same order at each time point.

At the end of the experimental period, all mice were sacrificed with CO_2_ and their intestinal tracts were harvested. The intestinal tracts were cut open and exposed to the air for 10 min, and then examined using bioluminescence imaging. In addition, stools from all animals were collected and weighed at 12 and 20 h post-inoculation, and again at the end of the experimental period. The number of *V. parahaemolyticus* contained in the stool samples was determined by plating a 10-fold serial dilution of the stool on TCBS agar (LandBridge Beijing, China) with and without kanamycin (50 μg/ml). *V. parahaemolyticus* appears as blackish green colonies on TCBS agar. Each group had 4 mice, and the experiment was repeated 3 times.

### Statistical analysis

Statistical analyses were performed using SPSS software version 17.0. Differences in the amounts of released LDH between the control group, the *lux*-expressing strain, and the parental strain were determined using one-way analysis of variance (ANOVA). Repeated measurement data analysis of variance (RMANOVA) was used to test the difference between the average luminous intensity of the control and treatment groups. A probability value of *p* < 0.05 was considered statistically significant.

## Results

### *B. fragilis* inhibits the growth of *V. parahaemolyticus in vitro*

In the Oxford cup assay, active ingredients contained in the liquid will diffuse freely into the agar, resulting in a zone of inhibition around the cup if there is antibacterial activity. Assays are conducted at a low temperature to allow time for the diffusion to occur.

**Figure 1:**
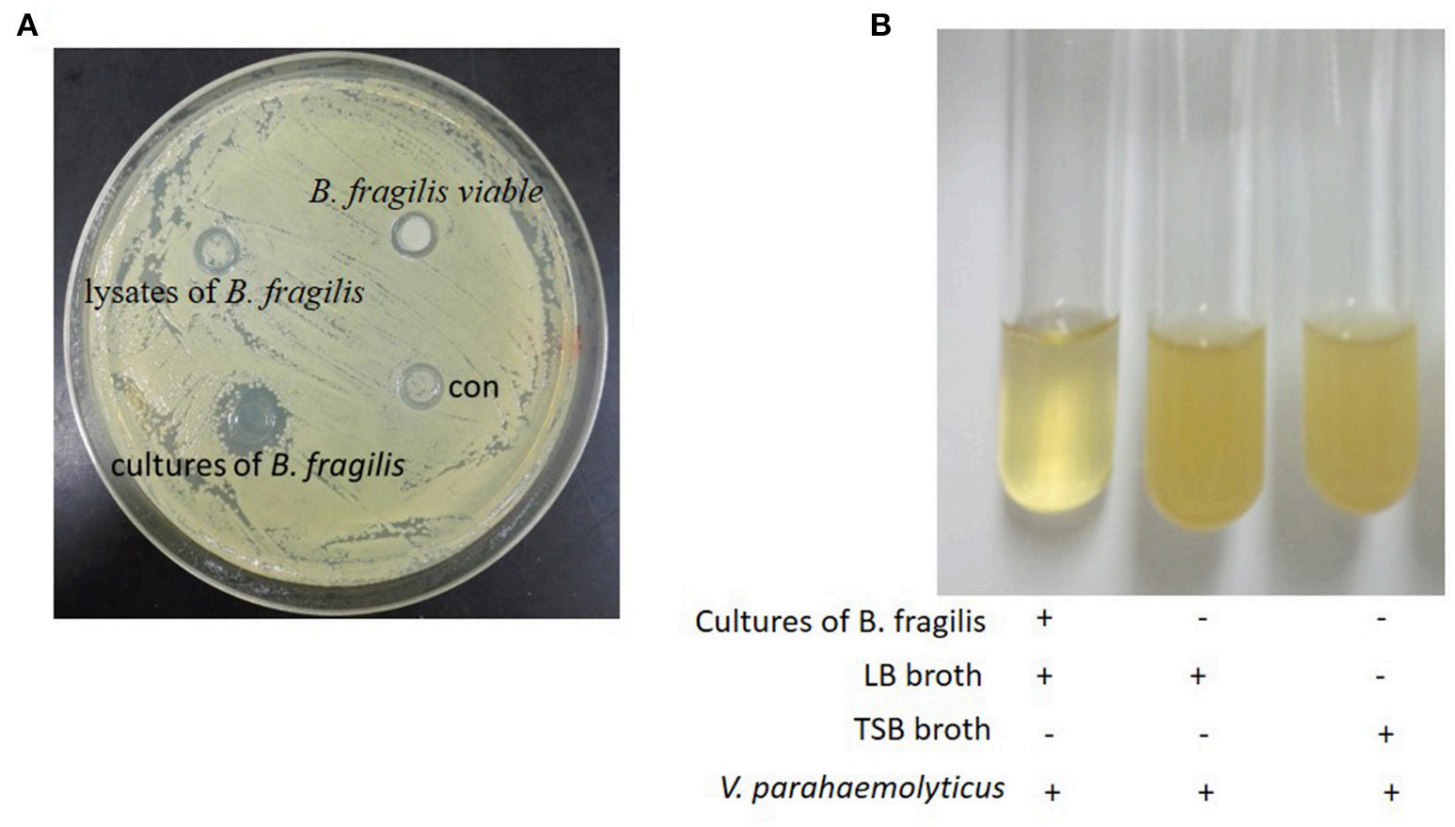
**The Oxford cup assay. (A)** The oxford cup assay of the cultures of *B. fragilis. V. parahaemolyticus* grew well on the LB agar but there was inhibition zone around the Oxford Cup with the culture of *B. fragilis* (the Oxford Cups were removed). **(B)** *B. fragilis* was cultured in TSB broth for 16 h and the 2.5 ml culture was mixed with 2.5 ml fresh LB broth. *V. parahaemolyticus* could grow well in LB broth and TSB broth after being cultured for 8 h, but it could not grow in the mixture of the LB broth and the culture of *B. fragilis*.

In our experiments, negative control strain *V. parahaemolyticus* grew both around and inside the Oxford cups. However, a zone of inhibition was observed around the cups containing *B. fragilis* culture. No zones of inhibition were observed around cups containing filtered *B. fragilis* lysate or resuspended bacteria (Figure 1A). To confirm these results, we cultured *V. parahaemolyticus* in LB broth with and without *B. fragilis* at a ratio of 1:1 by volume at 37°C anaerobically. After 8 h, the culture medium of the *B. fragilis* treatment remained clear, while the culture medium of the control was turbid (Figure 1B).

### *B. fragilis* protects intestinal cells and immune cells from damage caused by *V. parahaemolyticus*

*B. fragilis* was added to LoVo or RAW 264.7 cells in an attempt to simulate the relationship between normal intestinal epithelial cells or macrophages and intestinal bacteria. The cells were then infected with *V. parahaemolyticus* to explore whether *B. fragilis* could protect the cells from damage caused by the pathogen. RTCA and light microscopy were used to detect toxic effects of *V. parahaemolyticus* on the cells.

**Figure 2:**
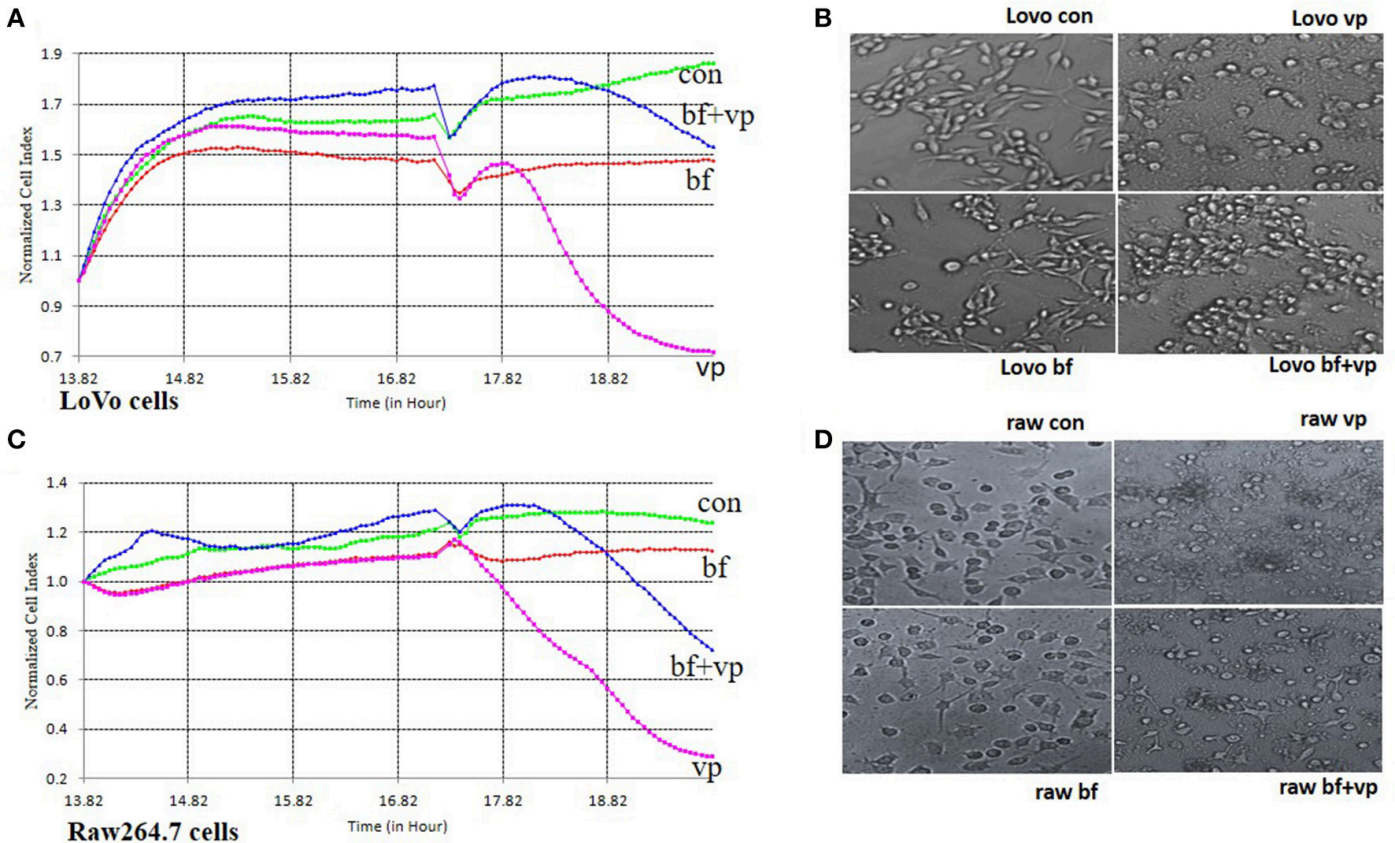
**RTCA pathogen inhibition assay. (A)** The RTCA Normalized Cell Index (NCI) curves of the LoVo cells co-incubated with medium (con), *B. fragilis* (bf), *V. parahaemolyticus* (vp), or both two bacteria (bf+vp). **(B)** The light morphology of the LoVo cells co-incubated with medium, *B. fragilis, V. parahaemolyticus*, or both two bacteria (200 ×). **(C)** The RTCA NCI curves of the Raw 264.7 cells co-incubated with medium, *B. fragilis, V. parahaemolyticus*, or both two bacteria. **(D)** The light morphology of the Raw 264.7 cells co-incubated with medium, *B. fragilis, V. parahaemolyticus*, or both two bacteria (200 ×). All the cells were cultured overnight and the cells of the bf+vp groups and bf groups were added with *B. fragilis* (MOI = 500) at about 14 h, *V. parahaemolyticus* (MOI = 50) was added to the cells of the bf+vp groups and vp groups at 17 h, the observation was finished after another 3 h. The NCI curves could represent the growth state of the cells during the whole time. The normal LoVo cells are spindle-shaped and the normal Raw264.7 cells are polygon as shown in **(B,D)** con group, when the cells were damaged, they became sparse, rounding and shedding as shown in **(B,D)** vp group, the bf group shaped like the con group and the bf+vp group shaped between the con group and the vp group.

Following incubation of LoVo or RAW 264.7 cells with *B. fragilis* (MOI = 500) for 8 h, the normal cell index (NCI) curves remained similar to those of the bacteria-free controls. However, the NCI curves of cells incubated with *V. parahaemolyticus* (MOI = 50) rapidly declined. The morphology of the cells infected with *B. fragilis* also remained very similar to that of the controls, while cells infected with *V. parahaemolyticus* became wrinkled and lysed indicating significant cytotoxicity. However, when the cells were infected with *V. parahaemolyticus* (MOI = 50) after being incubated with *B. fragilis* (MOI = 500) for 3 h, both the degree and the rate of decline of the NCI curves were reduced compared with the *V. parahaemolyticus*-only treated cells. In addition, the morphology of the *B. fragilis* pre-treated cells was much closer to that of untreated cells compared with the *V. parahaemolyticus*-treated cells (Figure 2).

### Construction of the bioluminescent *Vibrio parahaemolyticus* strain

**Figure 3:**
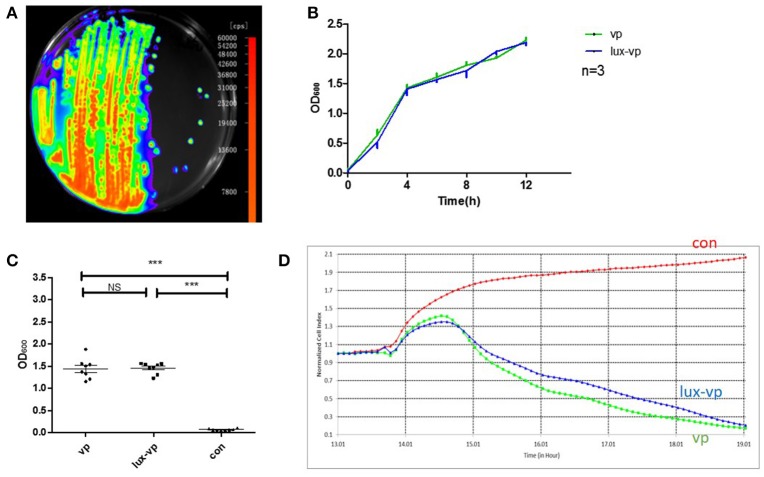
**Construction of the bioluminescent *Vibrio parahaemolyticus* strain. (A)** Luminescence imaging of the *lux*-expressing *V. parahaemolyticus*. **(B)** Growth curves of the wild type *V. parahaemolyticus* and its *lux*-expressing strain (Mean ± *SD, n* = 3). **(C)** The lactate dehydrogenase (LDH) released of lovo cells caused by the wild type *V. parahaemolyticus* and its *lux*-expressing strain compared with the normal lovo cells (^***^p < 0.001, One-way ANOVA). *Two* kinds of *V. parahaemolyticus* (MOI = 50) were added to the LoVo cells for 4 h and then the LDH released of lovo cells was detected. The more LDH released represented the more cells were damaged. **(D)** The RTCA NCI curves of the LoVo cells co-incubated with medium, the wild type *V. parahaemolyticus* (MOI = 50) and its *lux*-expressing strain (MOI = 50).

Plasmid pXEN-*lux*CDABE was successfully transformed into *V. parahaemolyticus* (Figure 3A). To determine whether the plasmid affected the growth and toxicity of the bacterium, we compared the growth rates and cellular toxicity of the *lux*-expressing and parental strains. The growth curves showed that the growth rate of the *lux*-expressing strain was similar to that of the wild-type strain (Figure 3B). In addition, there was no significant difference (*p* = 0.831) in the amount of released LDH between the two strains (Figure 3C). The NCI curve of LoVo cells incubated with the *lux*-expressing strain was also similar to that of cells infected with the wild-type strain (Figure 3D).

### *B. fragilis* may reduce the duration of *V. parahaemolyticus* infection in mice

**Figure 4:**
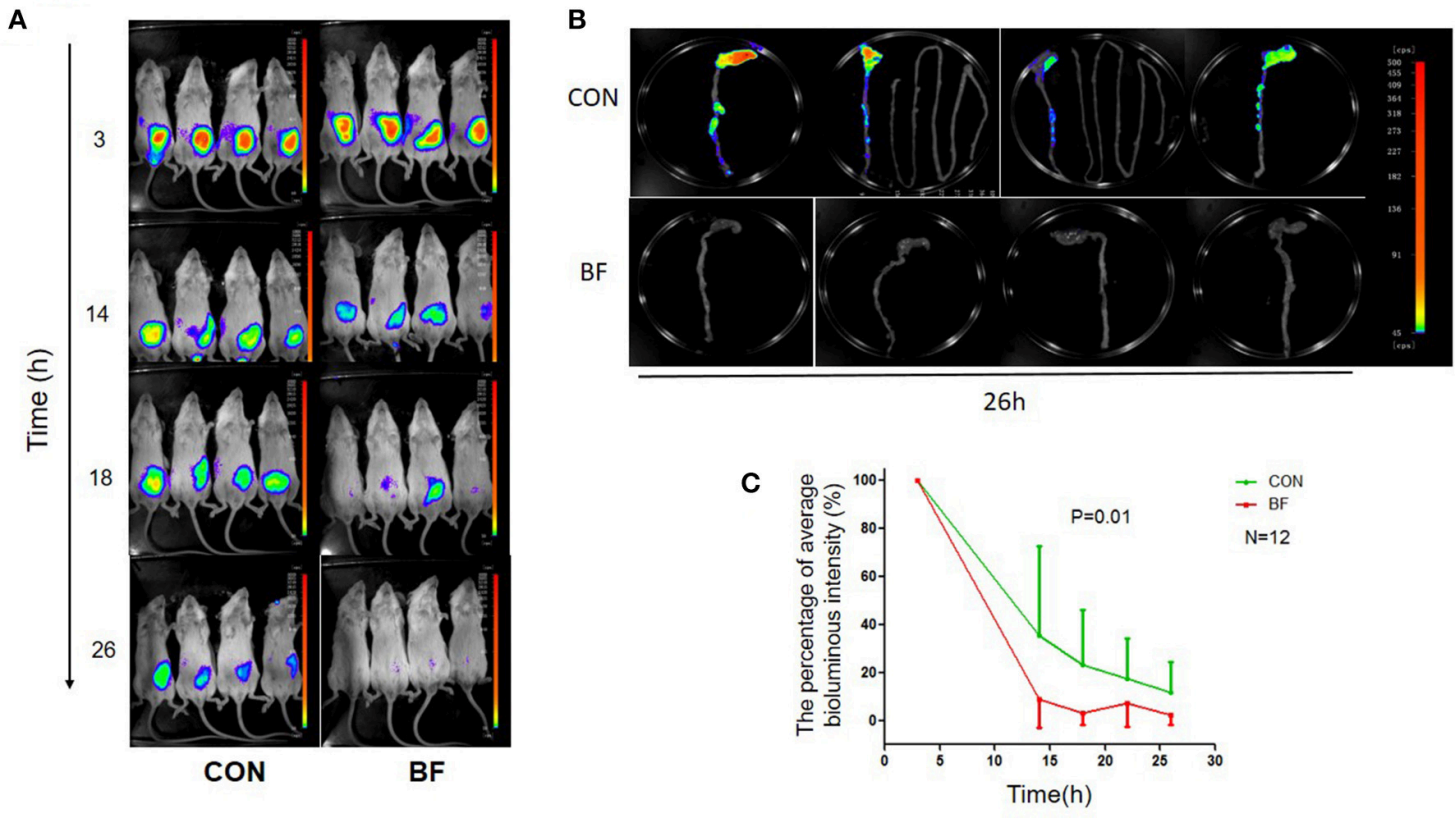
**Bioluminescence imaging of the mice. (A)** Bioluminescence imaging of BABL/c mice in the control group (CON) and the *B. fragilis* treatment group (BF) infected with the *lux*-expressing *V. parahaemolyticus*. **(B)** Bioluminescence imaging of BABL/c mice's intestinal tracts in the control group (CON) and the *B. fragilis* treatment group (BF). **(C)** The average luminous intensity percentage at various time points of the two groups (Mean ± *SD*, RMANOVA).

Using *in vivo* imaging, we traced the real-time proliferation and displacement of *lux*-expressing *V. parahaemolyticus* in mice. Following gavage with *lux*-expressing *V. parahaemolyticus* at a dose of ~10^10^ cfu, the luminous region and luminous intensity at 3 h post-infection were similar for all mice (Figure 4A). Half of the mice were then treated with *B. fragilis* at 3 and 14 h post-infection with *lux*-expressing *V. parahaemolyticus*. At 14 h post-infection, the luminous intensity of the mice in the treatment group was obviously weaker than that of the control group, and continued to decrease with time. By 26 h post-infection, luminescence in the treatment group was almost below the limit of detection, while the control group still had significant, although markedly decreased, bioluminescence.

At 26 h post-infection, all mice were sacrificed and their intestinal tracts were removed for bioluminescence imaging. Bioluminescence was undetectable in the intestinal tracts of mice from the treatment group, but was still evident in the intestinal tracts of animals from the control group. There was no bioluminescence in the small intestine of control mice, with the luminous regions located in the colon, especially the cecum (Figure 4B). RMANOVA analysis of the average percent luminous intensity between the two groups revealed that the luminous intensity of the treatment group was significantly decreased (*p* = 0.01) compared with the control (Figure 4C). The difference in luminous intensity could reflect the difference in the number of bacteria, we inferred that treatment with *B. fragilis* decreased *V. parahaemolyticus* colonization of the mouse intestine.

**Figure 5:**
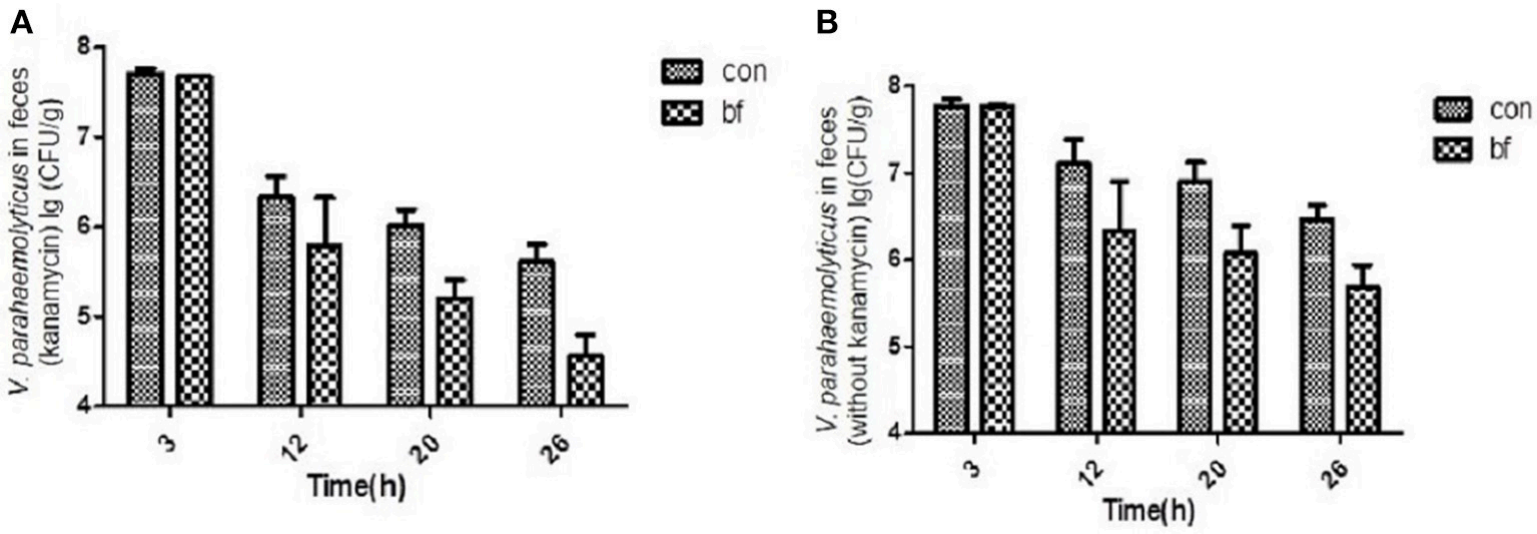
**The quantity of *V.parahaemolyticus* in mouse feces. (A)** The quantity of *V.parahaemolyticus* in mouse feces isolated with TCBS ager (kanamycin, 50 μg/ml) at various time points of the two groups. **(B)** The quantity of *V.parahaemolyticus* in mouse feces isolated with TCBS ager (without kanamycin) at various time points of the two groups.

The number of viable *V. parahaemolyticus* cells in stool samples collected from the two groups was determined using selective TCBS agar (with/without kanamycin, 50 μg/ml; Figure 5). During the experiment, some of the *V. parahaemolyticus* would lose the *lux* plasmid as well as the resistance to Kanamycin. Figure 5A showed the *V. parahaemolyticus* in the stool that still had the *lux* plasmid and Figure 5B showed all the *V. parahaemolyticus* in the stool. We found that the *V. parahaemolyticus* load in stools from the treatment group was significantly less than that of the control group, regardless of whether or not kanamycin was present in the medium.

## Discussion

*V. parahaemolyticus* is an important food-borne pathogen worldwide. Intestinal infections caused by bacterial pathogens are treated with antibiotics; however, there are three major drawbacks to the use of antibiotics: (1) drug-associated side effects, (2) development of antibiotic resistance, and (3) destruction of normal intestinal flora. The use of probiotics to treat intestinal infection would eliminate these problems. Most research about the antagonistic effects of other bacterial species on *V. parahaemolyticus* has focused on applications for aquaculture (Wu et al., 2014; Liu et al., 2015), and most of the strains used cannot be considered probiotics. Studies on the use of probiotics for treating intestinal infection caused by *V. parahaemolyticus* are rare, and only strains of *Lactobacillus* have been shown to inhibit *V. parahaemolyticus* both *in vitro* and *in vivo* in mice (Yang et al., 2013). In the current study, we identified a novel commensal *B. fragilis* strain (Deng et al., 2016) that could reduce cellular damage caused by *V. parahaemolyticus* and short the colonization period of *V. parahaemolyticus* in the intestines of mice.

In this study, the effects of *V. parahaemolyticus* was inhibited by *B. fragilis* coinfection *in vitro*; however, we did not explore which substances were responsible for the antimicrobial effect. According to Berger's Systemic Bacteriology Handbook (2nd Edn.), *B. fragilis* metabolites are rich in small organic acids, which might inhibit *V. parahaemolyticus*. The exact mode of inhibition should be the subject of further research.

RTCA allows continuous monitoring of the experiment, but only reflects the integrated state of the cells, while traditional endpoint methods (such as LDH analysis and morphological observation) only analyze certain aspects of the infection process, and not provide continuous data. Therefore, we combined RTCA and microscopic observation of cell morphology to monitor the toxicity of the different bacterial strains in the cells, providing richer, more accurate data. Following incubation of LoVo and RAW 264.7 cells with *B. fragilis* for 8 h, the RTCA NCI curves and cellular morphology showed no significant difference to the control, indicating that *B. fragilis* has no obvious cytotoxicity toward the human cells examined in this study. As a normal human intestinal symbiont, *B. fragilis* was first isolated from an abdominal abscess, and was considered a conditional pathogen for a long time (Mazmanian and Kasper, 2006). However, a large number of in-depth studies have shown that *B. fragilis* has a symbiotic relationship with the host in the intestine, and plays an important role in promoting host immune system maturation, inhibiting excessive inflammatory activity, and improving host intestinal flora structure (Mazmanian et al., 2008; Round et al., 2011; Hsiao et al., 2013). Polysaccharide A (PSA), a zwitterionic polysaccharide produced by *B. fragile*, has been extensively studied. It is reported that PSA is the main component of anti-inflammatory effects of *B. fragilis* (Mazmanian et al., 2005, 2008).

While our experiments demonstrated that *B. fragilis* has no obvious toxic effects on cells, the NCI curves of the LoVo and RAW 264.7 cells incubated with *V. parahaemolyticus* were significantly decreased within 2 h compared with that of the control. In addition, both cell types showed obvious morphological changes following infection, with cells first becoming round and then crumpled/lysed. These findings showed that *V. parahaemolyticus* was obviously toxic to both cell types. The major virulence factors of *V. parahaemolyticus* include capsular polysaccharide, hemolysin, and a type III secretion system. Capsular polysaccharide may help bacteria adhere to the intestinal cell surface, while type III secretion systems are used to inject toxins into cells, hemolysin has both cytotoxic and enterotoxic properties (Hsieh et al., 2003; Raghunath, 2014). When the various virulence factors of *V. parahaemolyticus* injure intestinal epithelial cells, the inflammatory substances released from intestinal tissue result in the migration of a variety of immune cells, including macrophages, to the site of infection (Waters et al., 2013). Macrophages play an important role in innate immunity, and as *V. parahaemolyticus* appears to attack and kill macrophages (Burdette et al., 2008), the immune response would be inhibited.

We also incubated *B. fragilis* with the LoVo cells to try to mimic the normal commensal relationship, and then added *V. parahaemolyticus*. RTCA and morphological observation showed that *B. fragilis* could protect LoVo cells from the damage caused by *V. parahaemolyticus*. Similar results were also observed using a RAW 264.7 cell model. In our experiments, *B. fragilis* not only protected the intestinal epithelial cells, but also lessened the damage to macrophages caused by *V. parahaemolyticus*. These results suggested that commensal colonization of *B. fragilis* in the intestine might have a protective effect during subsequent *V. parahaemolyticus* infection, mitigating damage to both normal intestinal epithelial cells and to immune cells that migrate to the site of infection.

For the animal experiments carried out in the current study, we introduced luminescent plasmid pXEN-*lux*CDABE into *V. parahaemolyticus* to generate a bioluminescent strain. Growth curves and cytotoxicity assays showed that the introduction of pXEN-*lux*CDABE had no significant effect on the growth or cytotoxicity of *V. parahaemolyticus*. Infant rabbits infected with *V. parahaemolyticus* develop diarrheal diseases that mimic the diseases in humans (Ritchie et al., 2012). Although, infant rabbits are indeed a suitable animal model for the study of *V. parahaemolyticus*, we still used mice for *in vivo* imaging experiment because infant rabbits could not tolerate anesthesia. When infected with the *lux*-expressing *V. parahaemolyticus* strain, mice that were treated with *B. fragilis* post-infection showed a decrease in luminous intensity compared with the control. Fecal *V. parahaemolyticus* counts were performed using specific TCBS agar with and without kanamycin for each stool sample. Kanamycin-supplemented selection plates showed that the *lux* plasmid was lost by a portion of bacteria during the experiment because of the lack of kanamycin selection *in vivo*. However, the number of viable *V. parahaemolyticus* cells in the feces of the *B. fragilis* treatment group was less than that of the control group at all of the time points, regardless of whether or not kanamycin was used. This indicated that *B. fragilis* could help remove *V. parahaemolyticus* from the intestinal tracts of mice.

In summary, our experiments showed that *B. fragilis* secretes substances that inhibit the growth of *V. parahaemolyticus*, reduces cellular damage caused by *V. parahaemolyticus*, and shortens the colonization period of *V. parahaemolyticus* in the intestines of mice. All of these results support the development of *B. fragilis* as a probiotic, which might aid in the clinical treatment of gastroenteritis caused by *V. parahaemolyticus* in the future.

## Author contributions

ZL and HD did the experiments, analyzed data, and wrote the manuscript; YZ and YT constructed the *lux*-expressing strain, analyzed data; XW and YH analyzed the data; YL and YW provided the *B. fragilis* strain ZY-312; RY contributed to revise the manuscript; YB designed experiments, analyzed data, and provided overall direction, FZ provided overall directions and contributed to revise the manuscript.

## Funding

This work was supported by National High Technology Research and Development Program 863 (No. 2015AA020702) and Science and Technology Program of Guangdong, China (No. 201504291330439 & 509186479222 & 509141742062).

### Conflict of interest statement

The property of the *Bacteroides fragilis* strain ZY-312 we used in the experiments belongs to Guangzhou ZhiYi biotechnology Co. Ltd. The authors declare that the research was conducted in the absence of any commercial or financial relationships that could be construed as a potential conflict of interest.
